# Cutting‐Edge Advancements in the Antibiotics‐Gut Microbiota‐Urinary Tumour Axis

**DOI:** 10.1111/cpr.70023

**Published:** 2025-03-17

**Authors:** Jie Wang, Dengxiong Li, Ruicheng Wu, Dechao Feng

**Affiliations:** ^1^ Department of Urology Institute of Urology, West China Hospital, Sichuan University Chengdu Sichuan China; ^2^ Division of Surgery & Interventional Science University College London London UK

## Abstract

Gut microbiota regulates urological tumors. Antibiotics induce dysbiosis, altering tumor progression/therapy: reducing carcinogen metabolism but impairing immunity. Specific bacteria enhance immune responses and combat endocrine resistance. Future research should unravel microbiota‐cancer links and develop microbiome‐targeted therapies to optimize outcomes while preserving diversity.

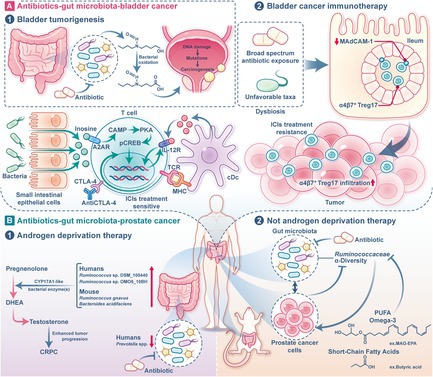

**Abbreviations**
ADT: androgen deprivation therapy
BBN: *N*‐butyl‐*N*‐(4‐hydroxybutyl)‐nitrosamine
BCa: bladder cancer
BCPN: *N*‐n‐butyl‐*N*‐(3‐carboxypropyl)‐nitrosamine
CRPC: castration‐resistant prostate cancer
FMT: faecal microbiota transplantation
ICIs: immune checkpoint inhibitors
PCa: prostate cancer
RCa: renal cancer
RCC: renal cell carcinoma

In recent years, the gut microbiota has emerged as a pivotal regulator of human health and disease. Beyond its well‐established roles in maintaining immune homeostasis and metabolic balance, the gut microbiome is increasingly recognised as a key player in cancer biology [1, 2, 3]. Urological tumours, including bladder cancer (BCa), renal cancer (RCa) and prostate cancer (PCa), are systemic diseases influenced by intricate molecular networks. While traditional research has primarily focused on genetic mutations, hormonal changes and environmental factors [4, 5, 6], growing evidence suggests that the gut microbiota contributes to the pathophysiology of these tumours through mechanisms involving immune modulation, inflammation and metabolic reprogramming [7, 8, 9].

The widespread use of antibiotics, though highly effective in managing infections, has significant and often unintended consequences for gut microbiota diversity and stability. This disruption, or dysbiosis, can lead to altered immune responses and systemic metabolic changes, potentially impacting the progression and treatment outcomes of urological tumours. For instance, antibiotic‐induced dysbiosis may exacerbate inflammation‐driven carcinogenesis or promote therapy resistance [10]. However, the precise mechanisms by which antibiotics influence the gut microbiota–tumour axis, particularly in the context of emerging therapeutic strategies such as immunotherapy, remain underexplored.

In a recent study published in *Nature*, Zimmermann et al. [11] highlighted the critical role of the gut microbiota in metabolising the carcinogen *N*‐butyl‐*N*‐(4‐hydroxybutyl)‐nitrosamine (BBN) and related nitrosamines in the development of BCa and other chemically induced tumours. It demonstrates that the microbiota generates carcinogenic metabolites, such as *N*‐n‐butyl‐*N*‐(3‐carboxypropyl)‐nitrosamine (BCPN), through deglucuronidation and oxidation reactions, altering their toxicokinetics and increasing exposure risk to bladder tissue. Antibiotics, by significantly reducing the gut microbiota, suppress the production of these metabolites and minimise exposure of urinary tissues, thereby markedly inhibiting the onset of BCa and the progression of invasive tumours. These findings underscore the pivotal role of the gut microbiota in the metabolism of chemical carcinogens and tumourigenesis while offering novel insights into personalised anti‐cancer strategies. Furthermore, in the immunotherapy of BCa, the use of antibiotics and the influence of the gut microbiota are also important. Zitvogel et al. [12] found that antibiotics, particularly when administered prior to immune checkpoint inhibitors (ICIs) treatment, disrupted gut microbial balance and reduced the expression of MAdCAM‐1 in intestinal venules, facilitating the migration of immunosuppressive α4β7+ Treg17 cells from the gut to tumour sites. This relocation compromises anticancer immune responses and diminishes the therapeutic effects of ICIs. The study further demonstrates that restoring gut microbiota through interventions like faecal microbial transplantation (FMT) or ectopic expression of MAdCAM‐1 can reverse antibiotics‐induced immunosuppression, underscoring the profound influence of gut dysbiosis on cancer immunotherapy outcomes. Interestingly, certain bacteria were found to enhance antitumour immunity and ICIs responsiveness. McCoy et al. [13] found that inosine, a metabolite produced by gut bacteria such as *Bifidobacterium pseudolongum* and *Akkermansia muciniphila* , could boost the efficacy of ICIs in mouse models. They propose that ICIs compromise gut barrier integrity, allowing inosine to enter systemic circulation and activate T helper 1 antitumour cells via adenosine 2A receptor pathways, ultimately promoting bladder tumour reduction. This finding provides new insights into how microbial metabolites may enhance immunotherapy outcomes. In brief, antibiotics influence gut microbiota to both reduce carcinogen metabolism to lower BCa risk, while potentially disrupting immune pathways and impairing treatment efficacy, whereas specific bacterial species and metabolites enhance antitumour immunity, highlighting the complex role of gut microbiota in BCa.

In PCa, recent studies have revealed that the gut microbiota plays a critical role in endocrine resistance. Androgen deprivation therapy (ADT) is a primary treatment for PCa, but many patients eventually progress to castration‐resistant prostate cancer (CRPC). Research has shown that specific gut commensal bacteria can convert androgen precursors into active androgens, thereby promoting the progression of CRPC. Pernigoni et al. [14] demonstrated in mouse models and human samples that ADT leads to the expansion of specific commensal bacteria capable of converting androgen precursors into active androgens, accelerating the development of CRPC. Eliminating the gut microbiota with antibiotics delayed the onset of CRPC, even in immunodeficient mice. Additionally, FMT from CRPC patients to prostate cancer‐bearing mice conferred resistance to castration, whereas FMT from hormone‐sensitive PCa patients helped control tumour growth. These findings suggest that the gut microbiota promotes endocrine resistance in CRPC by providing an alternative source of androgens. Thus, modulating the gut microbiota may offer a novel strategy to delay or reverse endocrine resistance in PCa. Moreover, a recent study [15] revealed that even in patients not undergoing ADT, the gut microbiota exhibited a complex association with PCa progression and treatment response. It reveals that reduced faecal microbiota diversity is associated with increased tumour volume and aggressiveness, and human FMT from PCa patients promoted tumour growth in mouse models, underscoring a microbiota‐PCa crosstalk. Dietary intervention with omega‐3 long‐chain fatty acids was found to inhibit tumour growth in mice and reduce cancer upgrades in patients undergoing radical prostatectomy, while also decreasing faecal levels of *Ruminococcaceae* and its metabolite butyrate. Elevated butyrate levels were linked to early metastatic PCa, suggesting that butyrate may serve as a key mediator in the gut microbiota‐PCa interaction. The family *Ruminococcaceae*, a key component of the gut microbiota, primarily resides within the mucosal biofilm layer and plays a crucial role in maintaining gut homeostasis and producing short‐chain fatty acids, particularly butyrate [16]. Butyrate exhibits context‐dependent effects on cancer progression, acting as both a tumour suppressor and promoter. In colorectal cancer, for example, butyrate has been shown to induce apoptosis, senescence, and differentiation in cancer cell lines, while promoting tumour development in animal models [17, 18]. Butyrate also exerts important immunomodulatory effects, including the induction of Foxp3+ regulatory T cells and inhibition of dendritic cell maturation [19]. While these anti‐inflammatory properties may be beneficial in inflammatory diseases, they can contribute to an immunosuppressive tumour microenvironment in cancers like PCa, where high intra‐tumoral Treg density is associated with poor outcomes. Recent studies suggest that high serum butyrate levels may correlate with resistance to immune checkpoint inhibitors, such as anti‐CTLA‐4 therapy, by promoting Treg activity [20]. In summary, these conflicting effects underscore the need for further research, particularly in non‐intestinal cancers like PCa.

In RCa, recent studies primarily focused on using metagenomic sequencing to analyse the clinical correlations among the antibiotic gut microbiome and treatment responses [21]. For example, a study [22] published in *Annals of Oncology* investigated the impact of antibiotics on the clinical outcomes of patients with advanced renal cell carcinoma (RCC) treated with ICIs. The results showed that antibiotic use was significantly associated with reduced efficacy of ICIs, including a higher risk of progressive disease (75% vs. 22%, *p* < 0.01), shorter progression‐free survival (1.9 vs. 7.4 months, HR 3.1, *p* < 0.01), and shorter overall survival (17.3 vs. 30.6 months, HR 3.5, *p* = 0.03). These findings suggest that antibiotics‐induced gut microbiota dysbiosis may negatively affect ICI outcomes, highlighting the potential for microbiome modulation as a strategy to improve immunotherapy efficacy in RCC patients. However, in‐depth exploration of the underlying mechanisms remains limited. Future research should focus on identifying specific microbial species or metabolic pathways that contribute to immunotherapy outcomes. This could pave the way for novel therapeutic strategies, such as targeted prebiotics, probiotics, or faecal microbiota transplantation, to restore gut microbial balance and optimise ICI efficacy.

In summary, the relationship between the gut microbiota and urinary tumours is an emerging area of research with promising implications for both cancer prevention and treatment. Figure 1 shows the antibiotics–gut microbiota–urinary tumour axis. Antibiotics, which are commonly used for treating infections in clinical practice, have a profound impact on the gut microbiota, enabling urinary cancer development and treatment outcomes. Despite the advancements in understanding the antibiotics–gut microbiota–urinary tumour axis, several limitations in the current body of research should be acknowledged. First, individual variations in gut microbiota composition and function, driven by factors such as genetics, diet and environmental exposures, pose a significant challenge in interpreting and generalising findings across studies. These variations may contribute to inconsistent results and highlight the need for personalised approaches in future research. Second, the heterogeneity in study designs, including differences in sample sizes, methodologies and populations, limits the ability to draw definitive conclusions. Standardised protocols and larger, well‐controlled studies are needed to address these issues. Finally, while significant progress has been made, many mechanistic details remain unclear, particularly regarding the specific microbial taxa or metabolites involved and their precise interactions with host physiology. Addressing these gaps will be critical for advancing our understanding of this complex axis. Future research must focus on understanding how different antibiotics affect both the gut microbiome and cancer treatment outcomes. The development of more targeted antibiotic therapies that minimise disruption to the microbiome could help avoid the negative effects of dysbiosis while still effectively treating infections. Such precision in antibiotic use could enhance the body's immune response, improve the effectiveness of cancer therapies and prevent the unintended consequences of broad‐spectrum antibiotics. Additionally, the microbiome could serve as a predictive tool for cancer therapy. Profiling the microbiome of cancer patients could provide insights into how antibiotics might influence treatment success, allowing for more personalised care. Future studies should also explore how antibiotics might be strategically used to enhance cancer treatment by promoting beneficial microbes or modifying the tumour microenvironment. Overall, as our understanding of the microbiome's role in cancer deepens, it will be essential to develop strategies that optimise the use of antibiotics in cancer patients, ensuring they do not hinder therapeutic responses while preserving microbial diversity critical for immune function and tumour suppression.

**FIGURE 1:**
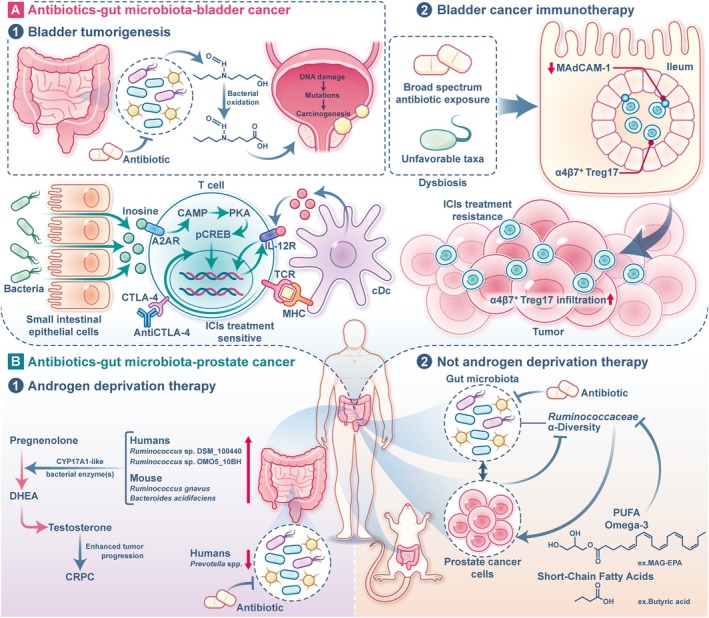
Antibiotics‐gut microbiota‐urinary tumour axis. (A) Antibiotics‐gut microbiota‐bladder cancer. In terms of bladder tumorigenesis, BBN is metabolised in the liver, where it is conjugated to glucuronide and excreted into the intestines. In the distal gut, β‐glucuronidase‐producing bacteria hydrolyze the glucuronide, releasing free BBN. Specific bacterial enzymes then oxidise BBN to form BCPN, a potent carcinogen. BCPN induces bladder carcinogenesis through DNA damage, promoting mutations that drive tumour formation. Antibiotics can disrupt the gut microbiota, altering this pathway and influencing bladder cancer risk. In terms of bladder cancer immunotherapy, broad‐spectrum antibiotic exposure leads to gut microbiota dysbiosis, characterised by unfavourable taxa and depletion of MAdCAM‐1 expression in the ileum. This facilitates the exodus of gut‐derived immunosuppressive Treg17 cells to tumours, weakening the ICIs therapy response. In addition, during ICIs immunotherapy (anti‐CTLA‐4 or anti‐PD‐L1), gut microbial species (such as *B. pseudolongum and A. muciniphila*) produce inosine, which activates A2AR signalling in T cells, promoting Th1/Tc1 differentiation and tumour infiltration. This process enhances *IL‐12Rβ2* and *IFNγ* transcription in T cells, driven by IL‐12 secretion from cDC cells, ultimately improving anti‐tumour immunity, while antibiotic‐induced dysbiosis impairs this response. (B) Antibiotics‐gut microbiota‐prostate cancer. In advanced prostate cancer patients undergoing ADT, gut microbiota composition is altered, leading to an accumulation of bacterial species capable of synthesising DHEA and testosterone from pregnenolone via CYP17A1‐like activity. These microbial‐derived androgens promote tumour growth and drive progression to castration‐resistant prostate cancer. In patients not undergoing ADT, antibiotics alter gut microbial diversity. Reduced gut microbiota alpha‐diversity correlates with increased prostate cancer burden, and faecal microbiota transplantation from high‐tumour patients promotes prostate cancer growth in mice. Omega‐3 MAG‐EPA supplementation reduces tumour progression by modulating gut microbiota, decreasing *Ruminococcaceae* abundance, and lowering faecal butyrate levels, highlighting a gut microbiome‐cancer crosstalk. BBN, *N*‐butyl‐*N*‐(4‐hydroxybutyl)‐nitrosamine; BCPN, *N*‐*n*‐butyl‐*N*‐(3‐carboxypropyl)‐nitrosamine; ICIs, immune checkpoint inhibitors; MAdCAM‐1, mucosal addressin cell adhesion molecule 1; cDC, conventional dendritic cells; ADT, Androgen deprivation therapy; DHEA, dehydroepiandrosterone.

## Author Contributions

J.W., DX.L. and RC.W. proposed the project, conducted a literature search, interpreted the data, and wrote the manuscript; all authors conducted data analysis and interpreted the data; DC.F. supervised the project and interpreted the data. All authors reviewed and edited the manuscript.

## Consent

The authors have nothing to report.

## Conflicts of Interest

The authors declare no conflicts of interest.

## Data Availability

Data sharing not applicable to this article as no datasets were generated or analysed during the current study.
